# Estimating the threshold of health workforce densities towards universal health coverage in Africa

**DOI:** 10.1136/bmjgh-2021-008310

**Published:** 2022-05-11

**Authors:** Adam Ahmat, James Avoka Asamani, Mourtala Mahaman Abdou Illou, Jean Jacques Salvador Millogo, Sunny C Okoroafor, Juliet Nabyonga-Orem, Humphrey Cyprian Karamagi, Jennifer Nyoni

**Affiliations:** 1 Health Workforce Unit, World Health Organization Regional Office for Africa, Brazzaville, Congo; 2 Health Financing Unit, World Health Organization Regional Office for Africa, Brazzaville, Congo; 3 Data and Knowledge Management Unit, World Health Organization Regional Office for Africa, Brazzaville, Congo

**Keywords:** Human Resources for Health, Threshold density, HRH planning, Health workforce density, Universal Health Coverage (UHC) service index, structural equation model, Reduced Gradient (GRG) approach, Africa

## Abstract

**Background:**

There have been past efforts to develop benchmarks for health workforce (HWF) needs across countries which have been helpful for advocacy and planning. Still, they have neither been country-specific nor disaggregated by cadre—primarily due to data inadequacies. This paper presents an analysis to estimate a threshold of 13 cadres of HWF density to support the progressive realisation of universal health coverage (UHC).

**Method:**

Using UHC service coverage as the outcome measure, a two-level structural equation model was specified and analysed in STATA V.16. In the first level of structural equations, health expenditure per capita—one of the cross-cutting inputs for UHC, was used to explain the critical inputs for service delivery/coverage. In the second level of the model, the critical inputs for service delivery were used to explain the UHC Service Coverage Index (UHC SCI), in which the contribution of the HWF was ‘partial out’.

**Results:**

The analysis found that a unit increase in the HWF density per 10 000 population is positively associated with statistically significant improvements in the UHC SCI of countries (β=0.127, p<0.001). Similarly, a positive and statistically significant association was established between diagnostic readiness and the UHC SCI (β=0.243, p=0.015). Essential medicines readiness was positively correlated but not statistically significant (β=0.053, p=0.658). Controlling for other variables, a density of 134.23 per 10 000 population across 13 HWF categories is necessary to attain at least 70% UHC SCI.

**Conclusion:**

Consistent with current knowledge, the HWF is a significant predictor of the UHC SCI. Attaining at least 70% of the UHC SCI requires about 134.23 health workers (a mix of 13 cadres) per 10 000 population.

**WHAT IS ALREADY KNOWN ON THIS TOPIC**

- In 2006 and 2016, WHO presented a minimum threshold density of 23 doctors, nurses and midwives per 10 000 population, required to attain at least 80% of skilled birth attendance as one of the indicators of the Millennium Development Goals, and 44.5 physicians, nurses and midwives per 10 000 population as the minimum that corresponds to the attainment of the median ranked country of selected tracer indicators of the SDGs, respectively, with the threshold densities focusing only on three cadres.

**WHAT THIS STUDY ADDS**

- Using recent data from 47 countries in the African region, this paper demonstrates that each dollar increase in current health expenditure per capita is associated with 3% improvements in the health workforce (HWF) density per 10 000 population, alongside improvements in other health system areas such as diagnostics, medicines availability.
- Attaining 70% or more of the universal health coverage (UHC) service coverage targets in the African region requires about 134.23 health workers (a mix of 13 cadres) per 10 000 population

**HOW THIS STUDY MIGHT AFFECT RESEARCH, PRACTICE AND/OR POLICY**

- The HWF density threshold estimated is directly linked to existing UHC indicators, taking into consideration 13 cadres of the HWF.
- Countries in the WHO African region should accelerate investments in health, especially in the HWF, towards a density of 134 per 10 000 to attain high UHC coverage scores.

## Background

Over the last two decades, the health workforce (HWF) has been at the top of the global health agenda but developing optimal and universally applicable threshold densities for the health HWF remained a challenge. There have been several efforts to establish normative global population ratios for HWF needs,^1–4^ but they have not addressed all the challenges and contexts. However, they have largely been successful in supporting advocacy that brought HWF crises, particularly in Africa, to the attention of policymakers at the global and regional levels. The WHO Global strategy on human resources for health^3 3^: has provided a basis for interventions to address the global HWF crises, especially in the African region. Nevertheless, the capacity and resources to develop and implement national strategies and to produce robust needs-based staffing remain a challenge. Therefore, countries continue to rely on established normative benchmarks and population ratios for planning. However, these are often neither country-specific nor disaggregated by cadre. Thus, it has become necessary to address these gaps and explicitly link the normative benchmarks to a routinely or periodically tracked measure of universal health coverage (UHC).

In 2006, WHO presented a minimum threshold density of 23 doctors, nurses and midwives per 10 000 population, required to attain at least 80% of skilled birth attendance as one of the indicators of the Millennium Development Goals.^4^ However, this threshold density was based on a single outcome variable, and its drawbacks have been highlighted as the focus of global health policy shifted to the more ambitious Sustainable Development Goals (SDGs), with UHC as the pivot target in health. Consequently, there have been several efforts to determine an ‘optimal’ threshold density of HWF at which the attainment of crucial health targets is plausible across countries.^1 2 5^ In 2014, the International Labour Organization (ILO) established the ‘staff access deficit’ indicator, a minimum threshold density of 34 doctors, nurses and midwives per 10 000 population (later revised to 41 per 10 000) for ensuring social protection.^2^ However, this has been questioned for an insufficient empirical link to health service coverage.^5^ Also, the report of the global initiative for ending maternal mortalities by 2035 determined that 59 physicians, nurses and midwives per 10 000 population are required to achieve lower than 50 maternal deaths per 100 000 live births.^1^ However, the lack of a clear policy linkage with the broader agenda of attaining UHC and the SDGs probably rendered this benchmark less popular under the current global health policy agenda.

In 2016, a ‘need-based’ methodology was developed by WHO known as the SDG-index, which seeks to attain the targets of at least 25% of some 12 SDG tracer indicators.^5^ ‘Need’ in this threshold was defined as the numbers of health workers required to achieve the median level of attainment (25%) of the selected tracer indicators of the SDGs. The approach produced a benchmark of 44.5 physicians, nurses and midwives per 10 000 population as the minimum that corresponds to the attainment of the median ranked country of selected tracer indicators of the SDGs. However, it only focuses on three cadres and without disaggregating the specific densities of each to allow identification of specific needs for planning at the country level. Also, it is understood that in developing the SDG index, the ‘decision to define need using the median level of attainment was made by an advisory committee’,^6^ for which the empirical basis is unclear.

The Regional Committee of Health Ministers in the WHO African Region in 2017 adopted a regional implementation framework for operationalising the Global strategy on human resources for health, which includes the SDG index as a milestone for countries. Since the attainment of the median ranked country of the tracer indicators will by no means represent the attainment of the objectives of UHC and the SDGs, it is essential to explore complementary ways by which the outcome and target of the HWF density threshold could be more intuitive, helpful in planning at country level, and specific as regards the densities needed for the different cadres of the HWF. To this end, the High-Level Consultative Group on HWF (HLG-HWF) of the WHO/ Africa Regional Office (AFRO) strongly recommended estimating the HWF density threshold that is directly linked to existing UHC indicators, taking into consideration the various cadres of the HWF of the national health systems.^7^

Therefore, this paper aims to estimate the threshold densities of the different HWF cadres towards UHC attainment in the WHO African region. Specifically, the paper partials out the contribution of the HWF density in the variations of UHC Service Coverage Index (SCI) in the African region using statistical models and determining the required density and mix of health workers for attaining various targets of UHC.

## Methods

The paper was guided by the input-side constructs of the UHC action framework of WHO/AFRO using the UHC SCI as a proxy measure of the coverage of essential health services, which was considered the most appropriate outcome indicator for the direct impact of improved access to health workers.^8^

### Conceptual framework

**Figure 1:**
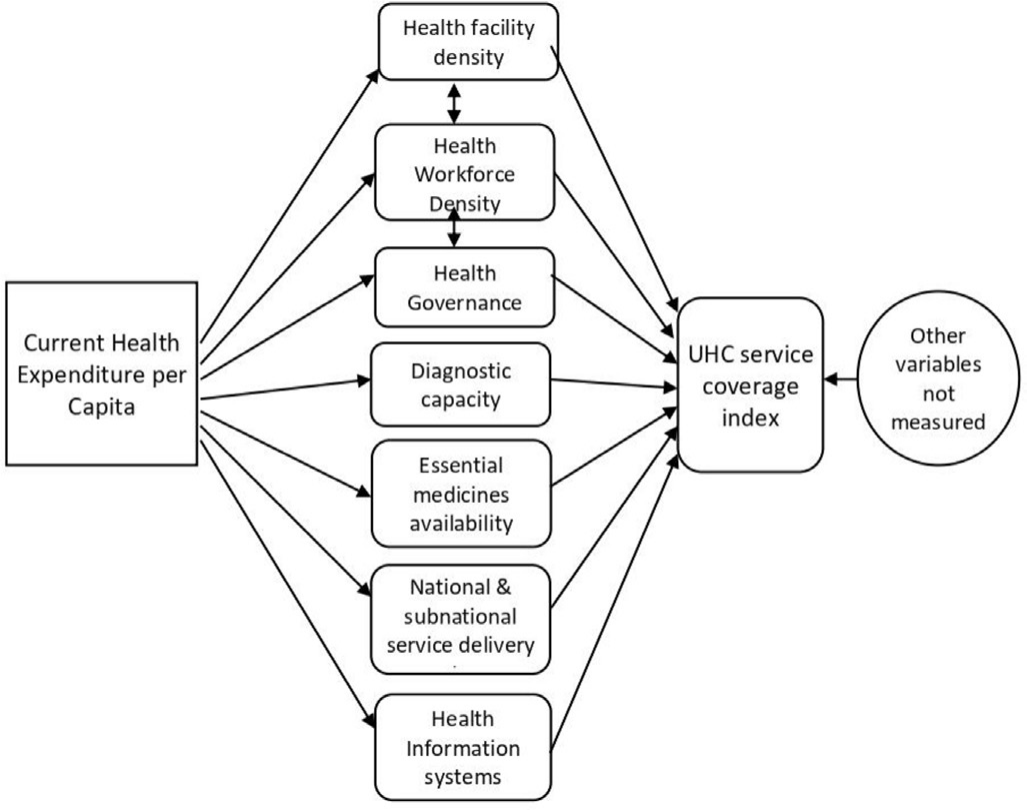
Structural relationship of the model for UHC service coverage index. UHC, universal health coverage.

Conceptually, the Africa Region recognises that health expenditure is undertaken across different investment areas, including: (1) HWF education and employment; (2) health infrastructure and equipment; (3) medical and diagnostic products; (4) information systems; (5) governance processes; (6) financial management systems and (7) service delivery systems.^9^ It is the country-specific breadth, depth and interactions across these investments that produce the health service coverage and its quality in a country (figure 1).^10 11^ Thus, attaining UHC is a function of the availability of an adequate and equitable distributed HWF; health infrastructure (health facilities); medicines, health products and technologies; and diagnostic capacities. These must also be underpinned by strong governance and efficient health financing mechanisms.^12–14^ Consistent with this well-known view, we present a simplified relationship in figure 1, in which we sought to empirically quantify the contribution of the HWF towards the health outcomes as measured by the coverage-related indices (service coverage scores) of the UHC index, by controlling for all the other potential variables for which data were available. The proposed conceptual relationships (figure 1) were tested empirically using available data from the 47 countries of the WHO African Region using a structural equation modelling procedure in STATA V.16.

### Definition of variables

The primary outcome variable and independent variables used in the empirical model are defined as follows:

- UHC SCI (UHC SCI) as the outcome variable, defined as the average coverage of essential services based on tracer interventions that include reproductive, maternal, newborn and child health, infectious diseases, non-communicable diseases (NCDs) and service capacity and access. The indicator is an index reported on a unitless scale of 0–100, computed as the geometric mean of 14 tracer indicators of health service coverage.^15^
- The independent variables are (1) the HWF density, which refers to the number of health workers across the standard 13 workforce categories (see table 1) per 10 000 population in a country (47 African countries); (2) the current health expenditure from both public and private sources per capita, defined as the average expenditure on health per person, measured in purchasing power parity—the purchasing power of national currencies against the international dollar; (3) the Essential medicines availability, defined as the proportion of essential medicines/products available in relation to the national essential medicines/products list; (4) the diagnostic capacity, measured as the proportion of needed diagnostic equipment and products available in health facilities compared with those in national health facility guidelines; (5) the health facility density (HFD) measured as the number of health facilities (of all types) that exist in a country per 1000 population.^16^

**Table 1:** List of health worker groups included in the analysis

ISCO-08 code	Health worker group
2211 and 2212	Medical doctors (generalists and specialists)
2221 and 2222, 3221 and 3222	Nursing and midwifery professionals and associates
2261 and 3251	Dentists and dental assistants, and therapists
2262 and 3213	Pharmacists, pharmaceutical technicians and assistants
3256	Medical assistants
3212	Medical and pathology laboratory technicians
3211	Medical imaging and therapeutic equipment technicians
2264 and 3255	Physiotherapists and physiotherapy technicians, and assistants
2267 and 3254	Optometrists and dispensing opticians
2240	Paramedical practitioners
2265	Dieticians and nutritionists
2263 and 3257	Environmental and occupational health and hygiene professionals
3253	Community health workers

ISCO, International Standard Classification of Occupations.

#### Data sources

All data used for the analysis were obtained directly from governments, WHO databases or publicly available sources and is aggregated on the integrated Africa Health Observatory,^17^ as shown in table 2.

**Table 2:** List of data sources and year

No	Variable	Data source	Years
1	Health facility density	Africa Health Observatory dataset for 2018 and 2019,^16^	2019
2	Health workforce density	State of the HWF in the African region: Survey report^27^	2018–2019
3	Current health expenditure per capita	Africa Health Observatory dataset for 2018 and 2019 16	2018–2019
4	Essential medicines availability	Harmonised Health Facility Assessments (HHFA) reports from countries Africa Health Observatory, WHO/AFRO	2018–2019
5	Diagnostic capacity	HHFA reports from countries Africa Health Observatory, WHO/Africa Regional Office (AFRO)	2018–2019
6	UHC Service Coverage Index	2019 UHC Monitoring report jointly published by WHO and the World Bank^28^	2019

HWF, health workforce; UHC, universal health coverage.

There was no routine and systematically collected data regarding the other variables of the conceptual framework, such as health governance, information systems and national/subnational service delivery systems across the countries; hence, they were not included in the empirical model. Structural equation modelling requires a large sample size, conservatively, in a ratio of at least ten to one variable included,^18–20^ but given that the WHO African Region has only 47 countries (the ‘sample’), it allowed for only a few variables to be included.

### Structural equation modelling procedures

#### Handling of missing data

There were missing data for some countries, which we first imputed for the missing data using multivariate imputation by chained equations (MICE) using R software.^21^ The regression equation 2019 UHC SCI is explained by the variables of overall HWF density (HWD), HFD, essential medicines readiness (EMR) and diagnostic readiness (Testing) (DRT). Based on this relationship, the missing variables were imputed using predictive mean matching (PMM). The MICE package in R software was used. PMM produces imputed values that resemble the observed values better than methods based on the normal distribution.^22^ This methodology ensures that if the original variable is right-skewed, PMM will then produce imputed values that follow the same distributional pattern. With that procedure, we generated datasets from which we proceeded to estimate the regression model parameters on each of the datasets and combined the estimates to one combined result, the resulting final dataset utilised in the analysis.

#### The structural equations

Based on the structural relationships illustrated in figure 1, two-level structural equations were specified in STATA V.16 SEM builder. The SEM approach was considered appropriate as it is best indicated in building indices based on an a priori conceptual framework. There are no predefined, tested and validated models for the exercise.^23^ In addition, it fits well to the investigation of causal inference hypotheses, calling for the use of several variables linked together by functional or structural relationships.^23^ It thus makes it possible to capture the direct and indirect effects of several types of variables, observable or latent. Finally, the SEM approach also offers the possibility of sequential or multilevel modelling, as proposed in the conceptual framework for this paper.

In the first level of the SEM, health expenditure per capita (HEC) was used to explain the level of some of the critical inputs for service delivery/coverage, given in equations 1–4:

(1) $$Health\;Workforce\;Density\;(HWD)=\alpha_{i}+\beta_{i}\times HEC+\epsilon_{i}$$

(2) $$Health\;Facility\;Density\;(HFD)=\alpha_{ii}+\beta_{ii}\times HEC+\epsilon_{ii}$$

(3) $$Essential\;Medicines\;Readiness\;(EMR)=\alpha_{iii}+\beta_{iii}\times HEC+\epsilon_{iii}$$

(4) $$Diagnostic\;Readiness\;(Testing)\;(DRT)=\alpha_{iv}+\beta_{iv}\times HEC+\epsilon_{iv}$$

Where α is the intercept (constant) for the relationship between the dependant and independent variables; β the slope of the regression line and *ε* is the error term or noise associated with each equation

In the second level of the SEM, the four inputs (equations HWF, HFD, EMR and DRT) were used as covariates to explain the UHC SCI (a standardised measure of 11 out of 14 tracer indicators of UHC covering RMNCH, infectious diseases and NCDs); the other indicators which constitute the service capacity and access component of the original UHC index were excluded because they were entered in the empirical model as covariates and could unduly inflate the degree of correlation if retained as part of the outcome variable. Our model was specified as equation 5 as follow:

UHC SCI = α_v_ + β_vi_ x HWF + β_vii_ x EMR +β_viii_ x DRT + β_iv_ x HFD + ε_v_ (5)

Where :

- α_v_ is the intercept (constant) of the equation
- β_vi_ is the slope of the regression line for the relationship between HWF and UHC SCI
- β_vii_ is the slope of the regression line for the relationship between EMR and UHC SCI
- β_viii_ is the slope of the regression line for the relationship between DRT and UHC SCI
- β_iv_ is the slope of the regression line for the relationship between HFD and UHC SCI
- ε_v_ is the error term or statistical noise associated with equations 5.

After implementing the primary analysis in STATA V.16, we used the resulting coefficients to fit equation 5 in Microsoft excel to simulate the HWF density required to attain different targets of the UHC SCI if the other variables are controlled. To simulate the most ‘optimal’ combination of the threshold densities of cadres of the HWF to attain various levels of the UHC SCI, we used a non-linear generalised reduced gradient (GRG) optimisation model, which was conducted using Microsoft Excel Solver.

#### Assessment of model fitness

After assessing the fitness of the model, the UHC SCI equation was used to simulate the population-weighted density of health workers that corresponds to various targets of the UHC SCI while controlling for the other covariates.

A χ^2^ test was used to evaluate the magnitude of discrepancy between the sample and the fitted covariance matrices (with the assumption that there will not be a statistically significant difference if there is a good model fit). The test was statistically insignificant (χ2=4.58, p=0.599), indicating a good model fit. The modelling literature suggests that a good model fit would provide a statistically insignificant chi-square result at a 0.05 alpha threshold.^18^ Overall, the model is reasonably fitted to the data with an average of 6% deviation; hence, the model was deemed appropriate in extrapolating a threshold of HWF density that corresponds to the attainment of various levels of the UHC SCI. However, one of the known limitations of this test is its low power to detect meaningful levels of model misspecification in small samples,^24^ such as the sample size used in the current analysis. Therefore, the root mean square error of approximation (RMSEA) was conducted, demonstrating how well a model contains the optimal parameters and fits the population’s covariance matrix.^19^ It is regarded as one of the critical fit indices due to its sensitivity to the number of estimated parameters in the model.^18 19^ The current model yielded an RMSEA value of 0.000 which shows that the model is a near-perfect fit for the data. As a rule of thumb, RMSEA ‘values less than 0.03 represent excellent fit’.^18^ The probability of a close fit for the current model is 0.70. Thus, the model explains 70% of the data.

This model predicts an average UHC SCI of 56.62 for the African Region as against the actual value in the UHC monitoring report of 2019, which was 56.44—thus indicating a mean absolute deviation of 0.183, which represents 0.32% of the value reported in the 2019 UHC monitoring report.

**Figure 2:**
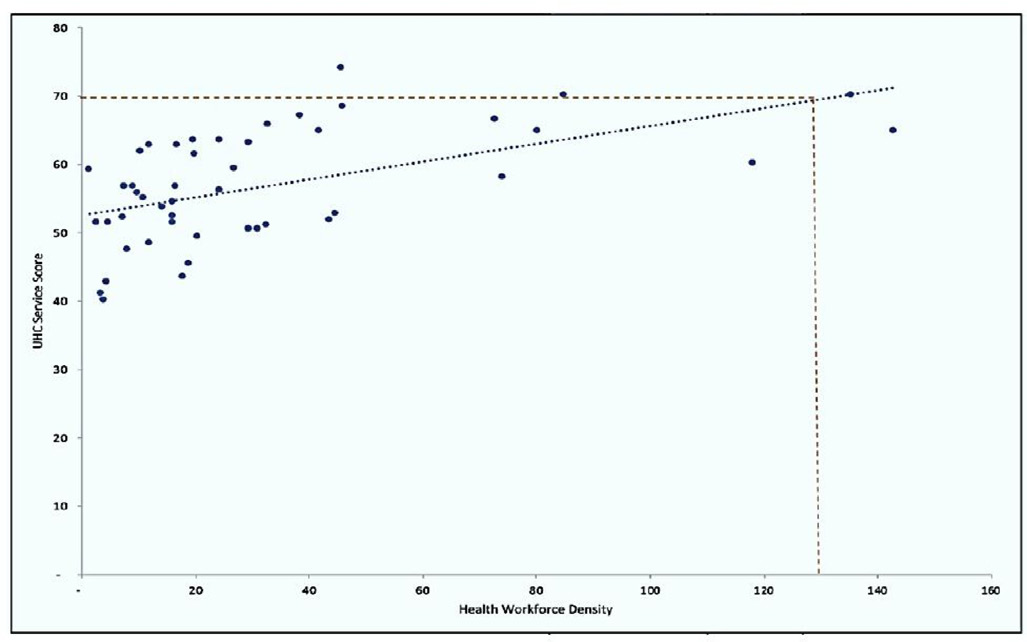
Density of 13 cadres of HWF per 10 000 population vs UHC SCI in 2019. HWF, health workforce; SCI, Service Coverage Index; UHC, universal health coverage.

As a cross-check of the estimated threshold, the UHC service coverage indices of the countries were plotted against their current aggregate HWF densities (comprising the 13 categories of HWF included in this analysis). In this single predictor analysis, a UHC SCI of 70%, 80% and 90% corresponded to approximately 136, 213 and 291 health workers per 10 000 population, respectively (figure 2).

#### Patient and public involvement

It was not appropriate or possible to involve patients or the public in the design, conduct, reporting or dissemination plans of our research.

## Findings

The first level of the analysis (table 3) shows that a country’s current HEC has a statistically significant positive relationship with the HWF density per 10 000 population (β=0.033, p=0.003). Hence, a US$1 increase in current HEC is associated with 0.033 improvements in the HWF density per 10 000 population. Current HEC also shows a positive effect, but not statistically significant, on diagnostic readiness (β=0.005, p=0.536), medicines readiness (β=0.014, p=0.104), and the number of health facilities per capita (β=0.00015, p=0.075). This result implies (without a good level of certainty) that a US$1 increase in HEC is associated with improvements in diagnostic readiness, EMR and the health facilities per capita at the rates of 0.005, 0.014 and 0.00015, respectively.

**Table 3:** Empirical relationships between the variables

Structural	Coef.β	SD	Z	P value	95% CI of β	
					Lower	Upper
Fist level of the analysis: equations 1–4						
Health workforce density per 10 000 population (1)						
Current health expenditure per capita	0.033*	0.011	2.930	0.003	0.011	0.055
Constant	19.731*	5.145	3.830	0.000	9.647	29.816
Health facility density per 10 000 population (2)						
Current health expenditure per capita	0.00015	0.00008	1.780	0.075	−0.00001	0.00031
Constant	0.329*	0.039	8.520	0.000	0.253	0.404
Essential medicines readiness (3)						
Current health expenditure per capita	0.014	0.009	1.620	0.104	−0.003	0.031
Constant	37.726*	3.276	11.520	0.000	31.305	44.146
Diagnostic readiness (4)						
Current health expenditure per capita	0.005	0.008	0.620	0.536	−0.011	0.022
Constant	39.296*	3.426	11.470	0.000	32.582	46.011
Second level of the analysis: Equation UHC SCI (5)						
2019 UHC service coverage index (5)						
Diagnostic readiness	0.243*	0.100	2.430	0.015	0.047	0.438
Health workforce density per 10 000 population	0.127*	0.033	3.870	0.000	0.063	0.192
Health facility density per 1000 population	2.235	4.694	0.480	0.634	−6.965	11.435
Essential medicines readiness	0.053	0.119	0.440	0.658	−0.180	0.286
Constant	39.762*	5.587	7.120	0.000	28.811	50.714

*Significant.

SCI, Service Coverage Index; UHC, universal health coverage.

The second level of the analysis shows that by holding the other variables constant, a unit increase in the HWF density per 10 000 population is positively associated with the UHC SCI, which is statistically significant (β=0.127, p=0.000). Also, there is a positive and statistically significant association between diagnostic readiness (β=0.243, p=0.015) and the UHC SCI. However, a positive but not statistically significant relationship between standardised HFD and the UHC SCI (β=2.235, p=0.634).

As a result, only the HWF density and diagnostic readiness made statistically significant and positive influences in explaining the UHC SCI variations across the African countries. One of the intents of this paper was to estimate the partial contribution of the HWF density to the variations of the UHC SCI in the African region. We used the findings (coefficients in table 3) to fit equation 5, to explore the prediction of the UHC SCI for a WHO/AFRO Member States as shown in equation 6:

**UHC SCI=0.127 x HWF +2.235 x HFD +0.053 x EMR +0.243 x DRT+39.762 …**. (6)

Using this model (equation 6) and by holding the other variables constant, a simulation of the HWF threshold densities was generated at various targets of UHC SCI, ranging from 60% to 90%, as shown in table 4. The simulation shows that between 60% and 70% of UHC service coverage, a unit increase in the coverage index target is associated with the need to increase HWF density by an average of 9.2%. For a UHC SCI above 70% and up to 80%, a unit increase in the coverage index is associated with the need to increase HWF density by an average of 4.7%. Similarly, a UHC SCI above 80% and up to 90% is associated with an average 3.2% increase in HWF density, and then an average of 2.4% increase in HWF density for any unit increase in the UHC SCI beyond 90%.

**Table 4:** Simulated targets of UHC service coverage index and the associated HWF threshold densities per 10 000 population

Targets (in %) of UHC service coverage index	HWF threshold density per 10 000 population	Marginal increase in HWF need (%)	Average marginal increase in HWF density
56	29.31	Baseline data (2019)	
60	55.77	–	
70	134.23	6.2	9.2
80	212.68	3.8	4.7
90	291.14	2.8	3.2

HWF, health workforce; UHC, universal health coverage.

### Simulating the optimal mix of HWF cadres

Using Solver add-in in Microsoft Excel and maintaining a non-linear GRG model, the model covariates were controlled, UHC service index constrained at 70% and the various categories of health workers included in the analysis were simultaneously varied to examine an ‘optimum mix’ at which the UHC target of 70% is attainable. The results in table 5 show a regional threshold density at 134.23 health workers per 10 000 population in the WHO African region, necessary to attain 70% of the UHC SCI. The results show disaggregation of this regional threshold density into various groups of health workers that together correspond to 70% of the UHC services index. For the attainment of that target of the UHC SCI, there is a need of a mix of 7.77 doctors per 10 000 population alongside 58.64 nurses and midwives per 10 000, 14.72 pharmacist and pharmacy technicians/assistants per 10 000 population, 14.0 medical and pathology laboratory scientists/technicians per 10 000 population, 25.34 community health workers per 10 000 population and 13.88 other cadres per 10 000 population.

**Table 5:** Mix of the health workforce threshold by health workers occupational group

ISCO-08 code	Health workers group	Current average regional density per 10 000 population	Density per 10 000 population for at least 70% UHC Services index	The proportion of the aggregate threshold
2211 and 2212	Medical Doctors (Generalists and Specialists)	3.29	7.77	5.8%
2221 and 2222, 3221 and 3222	Nurses and Midwives (Professionals and Associates)	13.99	58.64	43.7%
2261 and 3251	Dentists and dental technicians/assistants	0.74	5.28	3.9%
2262 and 3213	Pharmacist, Pharmacy Technicians/Assistants	1.28	14.72	11.0%
3256	Medical Assistants/Clinical Officers/Physician Assistants	0.28	0.90	0.7%
3212	Medical & Pathology Laboratory Scientists/technicians	1.25	14.00	10.4%
3211	Medical Imaging and therapeutic equipment operators	0.25	0.78	0.6%
2264 and 3255	Physiotherapists and physiotherapy assistants	0.28	0.91	0.7%
2267 and 3254	Optometrists and opticians	0.13	0.27	0.2%
2240	Paramedical practitioners	0.52	2.74	2.0%
2265	Dieticians and nutritionists	0.06	0.09	0.1%
2263 and 3257	Environmental and Occupational Health and Hygiene workers	0.54	2.92	2.2%
3253	Community Health Workers	6.69	25.20	18.8%
Health Workforce Density per 10 000 population		29.31	134.23	

ISCO, International Standard Classification of Occupations; UHC, universal health coverage.

## Discussion

There have been only a few attempts to define a cross country threshold for the needed HWF,^1–4^ which have not been without criticism on their practical feasibility and affordability, technical rigour and comprehensiveness in terms of covering most cadres of health workers^25^; hence there seems to be no full-proof optimal threshold, primarily due to data availability challenges and less standardisation of empirical models in the emerging field HWF planning.^26^ This analysis represents the first attempt to include most categories of health workers in developing a regional threshold density of HWF and for disaggregating the required density for the various cadres. It is also so far the only attempt (at least within the African Region) to directly derive a HWF threshold from the regularly calculated and reported UHC SCI.

As such, the model appears to be significantly sensitive to inconsistent, outdated or lack of data for the input variables. For instance, in countries with no recent estimates of EMR and diagnostic readiness, the model deviation has been considerable, ranging between −15% and +13%. Thus, updating the estimates as new and reliable data on the input variables become available is essential. Also, without health facility-level data, the scope and utility of analysis focused on being guided rough estimates for national-level analysis.

Nevertheless, this simulation has an added value for planning the skill mix of health workers. Our analysis shows that at least 44% of the overall density of health workers need to be nurses and midwives; 19% community-based health workers; 11% pharmacists, technicians and assistants; 10% laboratory scientists, technicians and assistants; and 6% doctors. When compared with the overall composition of the HWF in the African region, there are significant similarities with nurses and midwives, doctors and community health workers but marked differences with pharmacists, technicians and assistants, and laboratory scientists, technicians and assistants. For instance, a recent report shows that of 3.6 million health workers in the Africa region, 37% are nurses and midwives, 3% pharmacists, technicians and assistants, 10.4% laboratory scientists, technicians and assistants, 9% doctors and 14% community-based workers.^27^ Thus, our findings suggest that there is scope to optimise the exiting skill mix of the HWF, informed by some efficiency analysis and the role task-sharing will play in the HWF configuration.

Owing to the paucity of literature on the development of HWF density thresholds or composite indices that combine several categories of health workers, we compare our results with two essential works carried out by the WHO and ILO targeting three cadres (doctors, nurses and midwives).

First, the WHO Global Human Resources for Health (HRH) Strategy: workforce 2030, which was based on 12 SDG tracer indicators and their contribution to the global burden of disease,^6^ determined that countries need about 44.5 doctors, nurses and midwives per 10 000 population to reach the median ranked scores of the SDG tracer indicators. This current analysis shows that the density would be much higher (about three times higher) when the contribution of several other cadres of health workers is considered. Even when only doctors, nurses and midwives are considered, the current estimate is about 49% higher, that is, 66.41 for this analysis vs 44.5. In addition to methodological differences between the previous work (SDG-index) and the current work, the present analysis is based on only 47 countries in the WHO African Region, whereas the previous one was based on all countries for which data were available. Thus, while the current analysis may suffer from sample size limitations, the previous one also had to deal with considerable heterogeneity in country contexts, especially between high-income, middle-income and low-income countries across different continents.

In addition, the current analysis included a range of 13 cadres of health workers compared with the three main categories (doctors, nurses and midwives) included in the previous study. Thus, the current analysis complements previous WHO work in that it demonstrates that when the contribution of other categories of health workers (in addition to doctors, nurses and midwives) is taken into account, the progressive achievement of a universal healthcare system requires an even higher density of health workers.

Second, it is worth noting that the work of Bustreo *et al*^1^ showed that to reduce maternal deaths to 50 per 100 000 live births, countries would need a minimum of 59 midwives, nurses and doctors per 10 000 population. This demonstrates that depending on the methodology (model specification and variables included) and the outcome sought, varying threshold densities are likely to be produced. The current analysis focuses on the progressive achievement of UHC, which is broader than just reducing maternal mortality, and a minimum target for the UHC SCI can be set at 70% or 80%, which is more ambitious than the attainment of the median rank (25%) of the SDG tracer indicators used for benchmarking in the 2016 SDG index. Therefore, the higher HWF index (or threshold) estimated in this analysis remains reasonable to planning and advocacy, but specific for the context of the African region where the contribution of other cadres (besides doctors, nurses and midwives) to service delivery is enormous and needed to be explicitly taken into account.

It is also important to note that, depending on the context, some countries may need much more than the density threshold for a higher target than 70% UHC SCI, while others may need less to achieve the same level of UHC SCI. This implies that health system efficiency, especially in the deployment and utilisation of the HWF, would have a role in the attainment of UHC. It also suggests the possibility of improving the accuracy of the health worker density threshold estimation if data quality is improved. It is also essential to explore the possibilities of measuring and including other contextual factors such as health system governance and leadership, efficiency and distributive equity of HWF to improve the empirical and theoretical link.

### Limitations

The approach used represents one of the first attempts to use SEM to model the ‘optimal’ density of different categories of health workers for the attainment of UHC service coverage targets. However, it has some inherent limitations from both methodological and data perspectives. First, the relatively small sample size (47 countries) with some missing data constrained the extent to which sophisticated analysis and the number of variables could be included in the model. However, others have argued that a sample size of more than 30 could yield acceptable statistical estimates, more so, when the current analysis is based on an all-inclusive sampling approach, with data from all 47 Member States of the WHO African Region. Second, the exclusion of other variables known to influence the attainment of UHC is a noteworthy shortcoming of the analysis. In this respect, the model explained roughly 70% of the UHC SCI, leaving some 30% which can be explained by variables that were not measured in this model. For example, it is widely known that essential factors such as leadership and governance of the health systems, equity and efficiency in the distribution of the health workers, among others, are necessary catalysts for attaining UHC. However, at the time of analysis, there were no reliable datasets with metrics that measure these critical elements of health systems across most or all countries within the WHO African Region. The exclusion of these variables may be considered as a limitation, and efforts must be made to take them into account in future updates when such data become available. Also, accounting for other outcomes beyond UHC, such as health security, leadership capacity, health system efficiency, etc, was challenging to incorporate mainly due to the lack of systematically collected data on these variables across the countries.

## Conclusion

This analysis was done in response to WHO/AFRO’s HLG-HWF recommendation to establish a HWF density threshold that is directly linked to existing UHC indicators, taking into consideration the various cadres of the HWF of the national health systems.

The analysis found a regional threshold density of 134 health workers for 10 000 population are required to attain at least an average of 70% of the UHC SCI in the WHO African region. For the attainment of that target of the UHC SCI, there is a need of a mix of 7.77 doctors per 10 000 population, 58.64 nurses and midwives per 10,000, 14.72 pharmacist and pharmacy technicians/assistants per 10 000 population, 14.0 medical and pathology laboratory Scientists/technicians per 10 000 population, 25.34 community health workers per 10 000 population and 13.88 other health cadres per 10 000 population.

The estimated threshold densities of HWF for UHC provide the information needed for holistic planning for all cadres of the HWF needed for service delivery at all levels of care and in all sectors.

## Data Availability

Data are available on reasonable request.
